# Over-expression of COX-2 mRNA in colorectal cancer

**DOI:** 10.1186/1471-230X-14-1

**Published:** 2014-01-02

**Authors:** Hennie MJ Roelofs, Rene HM te Morsche, Bjorn WH van Heumen, Fokko M Nagengast, Wilbert HM Peters

**Affiliations:** 1 Department of Gastroenterology, Radboud University Nijmegen Medical Centre, Nijmegen, Netherlands

## Abstract

**Background:**

Cyclooxygenase-2 (COX-2, *PTGS2*) is an enzyme involved in the synthesis of prostaglandins and thromboxanes, which are regulators of biologic processes such as inflammation, cell proliferation and angiogenesis. COX-2 over-expression was reported in many (pre) malignant tissues, but data strongly vary and seem to depend on the methodology used.

**Methods:**

Normal colorectal mucosa and paired cancerous tissue from 60 patients with colorectal cancer was investigated for the levels of COX-2 mRNA by real-time quantitative Polymerase Chain Reaction (qPCR). COX-2 levels were expressed relative to either: tissue weight or levels of the housekeeping genes beta-2 microglobulin (*B2M*) and glyceraldehyde-3-phosphate dehydrogenase (*GAPDH*).

**Results:**

COX-2 mRNA levels, normalized with respect to tissue weight or mRNA levels of the housekeeping genes *B2M* or *GAPDH*, were over-expressed in 80%, 70% and 40% of the colorectal tumor tissues, as compared to the paired adjacent normal colorectal mucosa samples, respectively. Highest mRNA COX-2 ratios tumor/normal were measured when expressed per mg tissue (mean ratio 21.6). When normalized with respect to the housekeeping genes *B2M* or *GAPDH*, mean tumor/normal ratios were 16.1 and 7.5, respectively.

**Conclusion:**

Expression of COX-2 mRNA levels per mg tissue is most simple in comparison to normalization with respect to the housekeeping genes *B2M* or *GAPDH*. Levels of COX-2 mRNA are found over-expressed in almost 80% of the colorectal tumors, compared to paired adjacent normal colorectal mucosa, suggesting a role of COX-2 as a potential biomarker for cancer risk, whereas inhibitors of COX-2 could be of value in chemoprevention of colon cancer.

## Background

The development of colorectal cancer is a multistep process, where mutations may occur in the oncogenes *K*-*ras* and *APC*, or in the tumor suppressor gene *p53*, causing cell degeneration and uncontrolled cell proliferation. Cell proliferation is pivotal in tumorigenesis and cyclooxygenases (COXs) are important regulatory enzymes in this process. Cyclooxygenases catalyze the conversion of free arachidonic acid into prostaglandin H2, which is the precursor of other prostaglandins and thromboxanes. These regulatory compounds play a role in various biological processes such as cell proliferation, angiogenesis, immune function and inflammation, which are all crucial in the development or progression of neoplasms [1].

The human COX family consists of three members, COX 1–3. COX-1 is found in most tissues and plays a role in homeostasis of many physiologic processes. COX-3 is an alternative splice product of COX-1 and is involved in the regulation of pain and fever. COX-2 is an inducible enzyme, whose expression can be influenced by pro-inflammatory and mitogenic stimuli like cytokines and growth factors. COX-2 plays an important role in the development of metaplastic and dysplastic tissue, as well as in the development and progression of cancer, by involvement in the regulation of cell proliferation, cell transformation, tumor growth, tumor metastasis and invasion [1,2].

Over-expression of COX-2 has been associated with various premalignant and malignant lesions of epithelial origin, in particular in organs of the gastrointestinal tract [3-8]. Tumors with high levels of COX-2 seem to be more aggressive [5] and patients bearing those tumors had a significantly reduced survival [9]. In addition, after drug-induced suppression of COX-2 levels in laboratory animals subjected to entero-esophageal reflux, less animals developed esophageal adenocarcinoma [10]. This is of particular interest since more or less specific inhibitors of COX-2, such as various non-steroidal anti-inflammatory drugs (NSAIDs), have been or are being developed, that could have a role in the chemoprevention of gastrointestinal neoplasms [6]. Evidence is accumulating that a considerable reduction in the development of adenomatous polyps or colorectal cancer could be achieved in individuals regularly taking NSAIDs [6]. Although most NSAIDs do not specifically inhibit COX-2, the findings of over-expression of COX-2 in many (pre) malignant tissues, in combination with the reported beneficial effects of NSAIDs on cancer prevention, suggests that inhibition of COX-2 may be crucial in this process.

Since alterations in COX-2 levels may be pivotal in influencing the development of colorectal cancer [11], it is crucial that COX-2 levels can be estimated in a reliable way. Different techniques were used to investigate the COX-2 levels in colorectal neoplasms, yielding a large variety in outcomes. Semi-quantitative immunohistochemical staining for COX-2 was applied, thereby using different COX-2 antibodies [3,12-15]. Quantitative assay of COX-2 mRNA levels was performed [4,16-18], whereas both techniques were combined in some studies [12-15]. Large variations in the colorectal COX-2 expression levels were reported. In most studies, higher levels of COX-2 mRNA were demonstrated in the tumor, compared to the normal colorectal tissue [4,14,15,17,18], however sample sizes generally were small. In two larger studies, no elevated COX-2 mRNA levels in the tumor were reported [12,16]. Furthermore, in the studies where mRNA levels were assayed, most often no paired samples of normal and cancerous tissue from the same patient were investigated. In addition COX-2 mRNA levels were normalized with respect to different housekeeping genes.

The aim of the present study was to quantify the COX-2 mRNA levels in 60 paired samples of non-necrotic tumor tissue and corresponding normal colorectal mucosa, taken at least 5 cm distant from the tumor. The COX-2 mRNA levels will be expressed per mg tissue, as well as relative to the levels of two different housekeeping genes, beta-2 microglobulin (*B2M*) and glyceraldehyde-3-phosphate dehydrogenase (*GAPDH*).

## Methods

Normal colorectal mucosa and tumor tissue of 60 patients with sporadic colorectal cancer was included in this study. These tissues were obtained as rest material after surgical intervention and tissues were processed and assayed anonymously. Patients did not receive any treatment just before surgery. Rest material after resection for colorectal cancer was collected at the Department of Gastroenterology, Radboud University Nijmegen Medical Center (RUNMC), in the period 1999–2002. Immediately after resection, the tissue was cooled on ice and transported to the Pathology Department, where the pathologist inspected the tissue within 30 min. Representative specimens of macroscopically vital, non-necrotic tumor tissue and normal colorectal mucosa, taken at least 5 cm distant from the tumor, were obtained. Specimens were transported on ice to the laboratory and the tissue was washed with ice-cold phosphate buffered saline (PBS), cut in small pieces, frozen in liquid nitrogen and stored at −80°C until use. Ethical approval for the anonymous use of colorectal rest material was obtained by the RUNMC medical ethical review committee.

### Assay of COX-2 mRNA levels

Tissue was kept frozen while small samples (10–20 mg tissue) were cut off for mRNA analyses. From normal colon tissue, only mucosa was scraped off from the frozen samples. The normal- and tumor tissue samples were put into separate vials, weighed, and immediately thereafter 0.5 ml TRIzol (Life Technologies, Pailey, UK) was added. The within day variation of weighing tissues in the 10–50 mg range, using a Mettler AC100 precision weighing device (Mettler Instruments, Tiel, the Netherlands), was estimated to be 0.2-0.6%. Tissue was homogenized by 10 strokes with a Teflon pestle. RNA was isolated according to the instructions of the manufacturer (Life Technologies). Subsequently, 1 μg RNA was converted into cDNA, using the Roche Transcriptor cDNA synthesis kit, according to the instructions of the manufacturer (Roche Diagnostics, Almere, the Netherlands). Detection and quantification of mRNAs was performed by real-time quantitative PCR (qPCR) using the CFX96 Real-Time PCR Detection System (Bio-Rad Laboratories, Veenendaal, the Netherlands). Sequences of the primers were designed using Primer3 software (http://primer3.ut.ee/). Primers are: COX-2, 5’-CCGGGTACAATCGCACTTAT-3’ and 5’-GGCGCTCAGCCATACAG-3’ (Isogen Life Science, Maarssen, The Netherlands); GAPDH, 5’-GAAGGTGAAGGTCGGAGTCA-3’ and 5’-TTGAGGTCAATGAAGGGGTC-3’ (Isogen Life Science); B2M, 5’-ATGAGTATGCCTGCCGTGTG-3’ and 5’-CCAAATGCGGCATCTTCAAAC-3’ (Biolegio, Nijmegen, The Netherlands). Pairs of primers are designed to localize on different exons.

PCR products of COX-2 and GAPDH were detected with SYBR Green (Molecular Probes, Life Technologies, Bleiswijk, the Netherlands). For detection of B2M, a specific molecular beacon 5’-FAM-CGCGTCGTGGGATGGAGACATGTAAGCAGACGCG-Dabcyl-3’ was used (Biolegio). The annealing temperatures of the COX-2, GAPDH and B2M primers were 65.0˚C, 59.0˚C and 60.5˚C, respectively.

Specificity of the PCR products of COX-2, GAPDH and B2M was confirmed by melting curve analysis and agarose gel electrophoresis. All real time qPCR analyses were performed in triplicate.

### Statistics

Real time qPCR data were converted to linear data by calculating the 2^-Ct^ values when data are expressed per mg tissue, and by calculating the 2^-ΔCt^ values for B2M and GAPDH normalized data. Data are expressed as arbitrary units or as ratios of COX-2 expression (tumor/normal mucosa). Statistical analyses were performed using GraphPad Prism, version 4.00 (GraphPad Software Inc., La Jolla, CA, USA).

## Results

**Figure 1:**
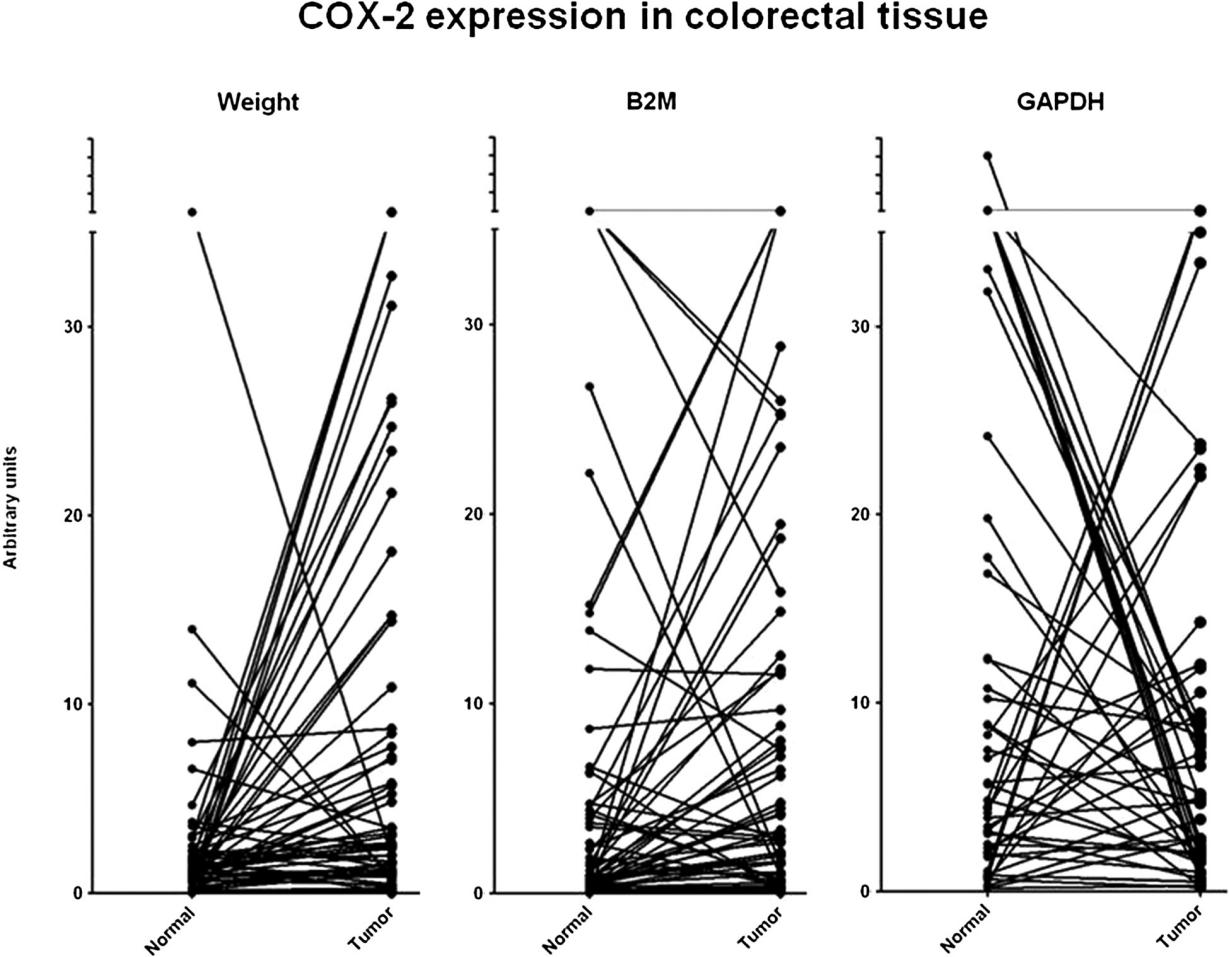
**COX**-**2 expression in colorectal tissue.**

Clinical data of the patients with colorectal cancer, who’s tissue was used in this study, are summarized in Table 1. Sixty paired samples of normal colorectal mucosa and corresponding tumor tissue were analyzed for COX-2 mRNA expression. Levels of COX-2 mRNA, measured in triplicate, were expressed per mg tissue weight or the expression was normalized with respect to mRNA levels of the housekeeping genes *B2M* and *GAPDH*. Mean expression of COX-2 mRNA was higher in colon tumor tissue, compared to the corresponding normal colorectal mucosa. However, COX-2 mRNA ratios tumor/normal clearly were dependent on the standardization used. Highest mean COX-2 ratios tumor/normal were observed when normalized with respect to tissue weight (mean ratio 21.6), followed by normalization with respect to the housekeeping genes *B2M* (mean ratio 16.1) or *GAPDH* (mean ratio 7.5); results are shown in Table 2. The COX-2 mRNA expression levels in normal colorectal mucosa and corresponding tumor tissue, normalized with respect to either tissue weight or the housekeeping genes *B2M* and *GAPDH*, are depicted in Figure 1.

**Table 1:** Characteristics of the patients with sporadic colorectal cancer

**Variable**	**Patients**
Number	60
Men/Women	34/26
Mean age (range)	65 (23–90) years
Tumor localization*	
-left colon	44
-right colon	16
Tumor stage**	
-T2	12
-T3	43
-T4	5
-lymph node metastases (N1/N2)	21 (12/9)
-distant metastases (M1)	5
Differentiation grade***	
-well	1
-moderate	56
-poor	2

*Left-sided colon: colon descendens, rectum and sigmoid; right sided colon: cecum, colon ascendens, colon transversum.

**Tumor staging was done according to the TNM classification.

***Note that for one tumor sample the differentiation grade was unknown.

**Table 2:** **Ratios of COX**-**2 mRNA expression in paired colorectal tumor**/**normal mucosa**, **normalized with respect to tissue weight or to the levels of the housekeeping genes *B2M *** **or *GAPDH***

	**Tissue weight**	**B2M**	**GAPDH**
Number of pairs	N = 60	N = 60	N = 55*
Mean ratio	21.6	16.1	7.5
Range	0.01-595.3	0.01-525.5	0.00-150.9
Ratio >1 (%)**	46 (77%)	42 (70%)	22 (40%)
Ratio <1 (%)***	14 (23%)	18 (30%)	33 (60%)

*B2M*, beta-2 microglobulin.

*GAPDH*, glyceraldehyde-3-phosphate dehydrogenase.

*Note that *GAPDH* was not detectable or the analysis did not succeed in 5 cases.

**Ratio > 1 means that COX-2 expression is higher in colorectal tumor tissue compared to paired normal mucosa.

***Ratio < 1 means that COX-2 expression is lower in colorectal tumor tissue compared to paired normal mucosa.

Ratios of COX-2 mRNA expression in tumor/normal colorectal mucosa, normalized with respect to tissue weight, correlated best with the corresponding ratios normalized for *B2M* (R = 0.952; P < 0.0001), and to a lesser extent with those normalized for *GAPDH* (R = 0.830; P < 0.0001). Ratios of COX-2 mRNA expression (tumor/normal) normalized with respect to tissue weight were not associated with gender (men vs. women, P = 1.0), tumor localization (left sided vs. right sided colon, P = 0.37), tumor grade (T2 vs. T3, P = 0.80; T2 vs. T3/T4, P = 0.57) or lymph node metastases (0 vs. N1/N2, P = 0.14).

## Discussion

COX-2 catalyzes the conversion of free arachidonic acid into prostaglandin H2, the precursor of other prostaglandins and thromboxanes. These compounds are pivotal in the regulation of cell proliferation, angiogenesis, immune function and inflammation, which may all contribute to the development and progression of neoplasms [1].

An important item in the measurements of COX-2 mRNA expression is the standardization. Standardization against tissue weight, with a variation of 0.2-0.6% in the weighing results when using a precision weighing device, is more accurate as compared to the reported variation of 0.7-5.3% for a quantitative real time PCR assay, by performing 3 repeats within one run [19].

Several studies reported that the COX-2 expression was elevated in colorectal tumors, in comparison to normal colorectal tissue. However, different techniques were used for estimating the COX-2 levels; semi-quantitative immunohistochemical staining of COX-2, making use of different COX-2 antibodies [3,12-15], quantitative PCR assay of COX-2 mRNA [4,16-18], or both techniques were applied simultaneously [12,14,15]. Immunohistochemical studies generally showed more staining in the tumor as compared to the normal colorectal mucosa, however staining positivity in the tumor varied from 35 to 62% in sporadic colorectal cancers [12,14] to 100% staining positivity in colorectal tumors from patients with familial adenomatous polyposis [13]. Variations in results on COX-2 mRNA analyses were also large. Again, in most studies COX-2 mRNA was found elevated in the tumor, however sample numbers were small [4,14,15,17,18]. In two larger studies, investigating 99 and 46 tumor samples respectively, no elevated COX-2 mRNA levels in the tumor were reported [12,16]. In most mRNA studies however, no paired samples of normal and cancerous tissue, obtained from the same patient, were investigated. In addition, COX-2 mRNA levels were normalized relative to the expression of one housekeeping gene, whereas the choice of which housekeeping gene was used also differed between the various studies.

Recently Carvalho et al. showed that COX-2 mRNA was up-regulated in an age-dependent fashion in colorectal carcinoma as compared to adenoma [20]. However, it needs to be further elucidated, whether the COX-2 levels in colorectal adenomas are elevated compared to normal mucosa. Eberhart et al. [4] reported elevated COX-2 levels in 3 of 6 colorectal adenomas compared with paired normal mucosa. Nosho et al. [14] found an over-expression of COX-2 mRNA in only 42% of the colorectal tumors investigated, being 63 adenomas and 27 carcinomas. In comparison with our data, where over-expression in 77% of colorectal carcinomas is noticed, this suggests that over-expression of COX-2 is lower in colorectal adenomas than in carcinomas, and that expression is increasing somewhere in the stage between adenoma and carcinoma. This could plead for the potential use of COX-2 mRNA levels in feces, as a potential non-invasive marker for colorectal cancer screening, as suggested by Kanaoka and co-workers [21,22].

For interpretation of quantitative mRNA measurements in clinical samples, a proper normalization is an essential component of reliable qPCR assays, which is necessary to correct for sample differences in cellular input, RNA quality and real-time efficiency [23]. According to recently published guidelines, normalization against a single reference gene is not acceptable [24], since large variations in the expression of commonly used housekeeping genes have been described [23-27]. For instance, in normal human colon samples, a more than 100 fold variation in GAPDH levels was reported; values varied between 8 × 10^4^ and 9.6 × 10^6^ copies of GAPDH per μg RNA [28]. In our study, COX-2 mRNA tumor/normal ratios normalized with respect to GAPDH showed the lowest correlation compared with normalization for tissue weight.

As stated by de Kok et al. in [23], ideally normalization could best be done based on the number of cells analyzed. In solid tissue, the amount of tissue is directly associated with cell numbers. Strikingly however, analysis based on cell numbers or amount of tissue was not applied in any of the recent studies on COX-2 mRNA expression in colorectal tissue [12,14-18].

We quantified the COX-2 mRNA levels per mg tissue, as well as relative to the levels of two different housekeeping genes *GAPDH* and *B2M*, in 60 paired samples of normal colorectal mucosa and corresponding non-necrotic tumor tissue. Detectable levels of COX-2 mRNA were found in all 60 paired specimens. After normalizing of the COX-2 levels relative to tissue weight, the COX-2 levels were higher in 77% of the carcinoma tissue compared to the adjacent normal mucosa, whereas the mean COX-2 levels in the tumor were 21.6 times higher compared to the normal mucosa levels. Since it is very simple and accurate to determine the tissue weight just before RNA extraction, this may be the method of choice, since there is only a small weighing error (0.2-0.6%) when using a precision weighing device.

Comparing the mRNA expression of the two commonly used housekeeping genes *B2M* and *GAPDH*, *B2M* seems the second best choice for normalization in colorectal tissue, since there is very much variation in the expression of *GAPDH* in normal colorectal tissue, as already outlined above [28].

## Conclusions

COX-2 mRNA levels as quantified by qPCR and normalized with respect to tissue weight, are over-expressed in 77% of colorectal carcinomas compared to adjacent normal colorectal mucosa, and mean carcinoma levels are almost 22 times higher than normal mucosa levels. Normalization with respect to tissue weight is simple and accurate, compared to normalization based on the levels of the housekeeping genes *B2M* or *GAPDH*. The common over-expression of COX-2 in colorectal carcinoma suggests a role for COX-2 as a colorectal carcinoma risk biomarker, whereas attention should also be focussed on COX-2 inhibitors, as potential promising chemopreventive drugs for colorectal cancer.

## Competing interests

The authors declare that they have no competing interests.

## Authors’ contributions

HMJR, RHMtM, BWHvH, FMN and WHMP designed the study; HMJR and RHMtM performed the assays; HMJR and WHMP wrote the first draft and all authors read, corrected and approved the final draft.

## Pre-publication history

The pre-publication history for this paper can be accessed here:

http://www.biomedcentral.com/1471-230X/14/1/prepub

## References

[B1] Chandrasekharan NV Simmons DL The cyclooxygenases Genome Biol 2004 5 241 10.1186/gb-2004-5-9-241 15345041 PMC522864

[B2] Chandrasekharan NV Dai H Roos KL Evanson NK Tomsik J Elton TS Simmons DL COX-3, a cyclooxygenase-1 variant inhibited by acetaminophen and other analgesic/antipyretic drugs: cloning, structure and expression Proc Natl Acad Sci U S A 2002 99 13926 13931 10.1073/pnas.162468699 12242329 PMC129799

[B3] Sano H Kawahito Y Wilder RL Hashiramoto A Mukai S Asai K Kimura S Kato H Kondo M Hla T Expression of cyclooxygenase-1 and −2 in human colorectal cancer Cancer Res 1995 55 3785 3789 7641194

[B4] Eberhart CE Coffey RJ Radhika A Giardiello FM Ferrenbach S DuBois RN Up-regulation of cyclooxygenase 2 gene expression in human colorectal adenomas and adenocarcinomas Gastroenterology 1994 107 1183 1188 7926468 10.1016/0016-5085(94)90246-1

[B5] Fujimura T Ohta T Oyama K Miyashita T Miwa K Role of cyclooxygenase-2 in the carcinogenesis of gastrointestinal tract cancers: a review and report of personal experience World J Gastroenterology 2006 12 1336 1345 10.3748/wjg.v12.i9.1336 PMC4124307 16552798

[B6] Brown JR DuBois RN COX-2: a molecular target for colorectal cancer prevention J Clin Oncol 2005 23 2840 2855 15837998 10.1200/JCO.2005.09.051

[B7] Mehta S Boddy A Johnson T Rhodes M Systematic review: cyclo-oxygenase-2 in human oesophageal adenocarcinogenesis Aliment Pharmacol Ther 2006 24 1321 1331 10.1111/j.1365-2036.2006.03119.x 17059513

[B8] Liu X Li P Zhang ST You H Jia JD Yu ZL COX-2 mRNA expression in esophageal squamous cell carcinoma (ESCC) and effect by NSAID Dis Esophagus 2008 21 9 14 18197933 10.1111/j.1442-2050.2007.00697.x

[B9] Buskens CJ Van Rees BP Sivula A Reitsma JB Haglund C Bosma PJ Offerhaus GJ Van Lanschot JJ Ristimäki A Prognostic significance of elevated cyclooxygenase-2 expression in patients with adenocarcinoma of the esophagus Gastroenterology 2002 122 1800 1807 10.1053/gast.2002.33580 12055587

[B10] Buttar NS Wang KK Leontovich O Westcott J Pacifico RJ Anderson MA Krishnadeth KK Lutzke LS Burgart LJ Chemoprevention of esophageal adenocarcinoma by COX-2 inhibitors in an animal model of Barrett’s esophagus Gastroenterology 2002 122 1101 1112 10.1053/gast.2002.32371 11910360

[B11] Greenhough A Smartt HJ Moore AE Roberts HR Williams AC Paraskeva C Kaidi A The COX-2/PGE2 pathway: key roles in the hallmarks of cancer and adaptation to the tumour microenvironment Carcinogenesis 2009 30 377 386 10.1093/carcin/bgp014 19136477

[B12] Antonacopoulou AG Tsamandas AC Petsas T Liava A Scopa CD Papavassiliou AG Kalofonos HP EGFR, HER-2 and COX-2 levels in colorectal cancer Histopathology 2008 53 698 706 10.1111/j.1365-2559.2008.03165.x 19102009

[B13] Brosens LA Keller JJ Pohjola L Haglund C Morsink FH Iacobuzio-Donahue C Goggins M Giardiello FM Ristimäki A Offerhaus GJ Increased expression of cytoplasmic HuR in familial adenomatous polyposis Cancer Biol Ther 2008 7 424 427 10.4161/cbt.7.3.5417 18094611

[B14] Nosho K Yoshida M Yamamoto H Taniguchi H Adachi Y Mikami M Hinoda Y Imai K Association of Ets-related transcriptional factor E1AF expression with overexpression of matrix metalloproteinases, COX-2 and iNOS in the early stage of colorectal carcinogenesis Carcinogenesis 2005 26 892 899 15695237 10.1093/carcin/bgi029

[B15] Loukanov T Kirilov M Fürstenberger G Müller-Decker K Localization of cyclo-oxygenase-2 in human recurrent colorectal cancer Clin Invest Med 2010 33 E22 E29 20144265 10.25011/cim.v33i1.11834

[B16] Gustafsson A Hansson E Kressner U Nordgren S Andersson M Wang W Lönnroth C Lundholm K EP1-4 subtype, COX and PPAR gamma receptor expression in colorectal cancer in prediction of disease-specific mortality Int J Cancer 2007 121 232 240 10.1002/ijc.22582 17290397

[B17] Delage B Rullier A Capdepont M Rullier E Cassand P The effect of body weight on altered expression of nuclear receptors and cyclooxygenase-2 in human colorectal cancers Nutr J 2007 6 20 10.1186/1475-2891-6-20 17767717 PMC2018695

[B18] Jahns F Wilhelm A Jablonowski N Mothes H Radeva M Wölfert A Greulich KO Glei M Butyrate suppresses mRNA increase of osteopontin and cyclooxygenase-2 in human colon tumor tissue Carcinogenesis 2011 32 913 920 10.1093/carcin/bgr061 21459756 PMC3314284

[B19] Pfaffl MW Georgieva TM Georgiev IP Ontsouka E Hageleit M Blum JW Real-time RT-PCR quantification of insulin-like growth factor (IGF)-1, IGF-1 receptor, IGF-2, IGF-2 receptor, insulin receptor, growth hormone receptor, IGF-binding proteins 1, 2 and 3 in the bovine species Domest Anim Endocrinol 2002 22 91 102 10.1016/S0739-7240(01)00128-X 11900967

[B20] Carvalho B Sillars-Hardebol AH Postma C Mongera S Droste JT Obulkasim A van de Wiel M van Criekinge W Ylstra B Fijneman RJ Meijer GA Colorectal adenoma to carcinoma progression is accompanied by changes in gene expression associated with ageing, chromosomal instability, and fatty acid metabolism Cell Oncol 2012 35 53 63 10.1007/s13402-011-0065-1 PMC3308003 22278361

[B21] Hamaya Y Yoshida K Takai T Ikuma M Hishida A Kanaoka S Factors that contribute to faecal cyclooxygenase-2 mRNA expression in subjects with colorectal cancer Br J Cancer 2010 102 916 921 10.1038/sj.bjc.6605564 20145612 PMC2833255

[B22] Kanaoka S Yoshida K Miura N Sugimura H Kajimura M Potential usefulness of detecting cyclooxygenase 2 messenger RNA in feces for colorectal cancer screening Gastroenterology 2004 127 422 427 10.1053/j.gastro.2004.05.022 15300574

[B23] de Kok JB Roelofs RW Giesendorf BA Pennings JL Waas ET Feuth T Swinkels DW Span PN Normalization of gene expression measurements in tumor tissues: comparison of 13 endogenous control genes Lab Invest 2005 85 154 159 10.1038/labinvest.3700208 15543203

[B24] Bustin SA Benes V Garson JA Hellemans J Huggett J Kubista M Mueller R Nolan T Pfaffl MW Shipley GL Vandesompele J Wittwer CT The MIQE guidelines: minimum information for publication of quantitative real-time PCR experiments Clin Chem 2009 55 611 622 10.1373/clinchem.2008.112797 19246619

[B25] Caradec J Sirab N Revaud D Keumeugni C Loric S Is GAPDH a relevant housekeeping gene for normalisation in colorectal cancer experiments? Br J Cancer 2010 103 1475 1476 10.1038/sj.bjc.6605851 20859286 PMC2990595

[B26] Barber RD Harmer DW Coleman RA Clark BJ GAPDH as a housekeeping gene: analysis of GAPDH mRNA expression in a panel of 72 human tissues Physiol Genomics 2005 21 389 395 10.1152/physiolgenomics.00025.2005 15769908

[B27] Stürzenbaum SR Kille P Control genes in quantitative molecular biological techniques: the variability of invariance Comp Biochem Physiol B Biochem Mol Biol 2001 130 281 289 10.1016/S1096-4959(01)00440-7 11567890

[B28] Bustin SA Absolute quantification of mRNA using real-time reverse transcription polymerase chain reaction assays J Mol Endocrinol 2000 25 169 193 10.1677/jme.0.0250169 11013345

